# Residual triamcinolone acetonide at macular hole after vitreous surgery

**DOI:** 10.4103/0301-4738.62650

**Published:** 2010

**Authors:** Atul Kumar, Subijay Sinha, Anoop Gupta

**Affiliations:** Dr. Rajendra Prasad Centre for Ophthalmic Sciences, AIIMS, New Delhi-11 00 29, India

**Keywords:** Internal limiting membrane peeling, macular hole, triamcinolone

## Abstract

Post traumatic macular holes have shown successful anatomic outcomes with vitrectomy with internal limiting membrane (ILM) peeling and gas injection. Intraocular use of triamcinolone acetonide (TA) crystals is gaining popularity in patients for visualization of the vitreous cortex, posterior vitreous detachment induction and ILM peeling during macular hole surgery. However, the possibility of residual steroid crystals clogging the hole at the conclusion of surgery exists. In our case, residual TA was observed biomicroscopically in the fovea on the seventh day after surgery, Optical Coherence Tomography (OCT) image of the eye showed a hyper reflective mass corresponding to the TA. However, a repeat OCT carried out four weeks after surgery showed recovery of the foveal morphologic features to an almost normal depression, with closure of the hole. Residual TA crystals in the macular hole post vitreous surgery may not interfere with ultimate macular hole closure or visual improvement.

Post traumatic macular holes have shown successful anatomic and functional outcomes with vitrectomy with internal limiting membrane (ILM) peeling and gas injection. Visual improvement of at least two Snellen lines has been reported in 69-94% of cases.[1] To improve anatomic outcomes after macular hole surgery, removal of the ILM has become widely prevalent. Indocyanine green (ICG) facilitates ILM peeling because it is stained extremely well with ICG. However, the use of ICG to stain ILM during macular hole surgery (MHS) has shown potential toxicity to photoreceptors causing irreversible visual loss. The dye is also photosensitive and persists in the retina and the optic nerve head for months after surgery.

Triamcinolone acetonide (TA) has been used recently for ILM peeling in patients requiring MHS.[2] TA is a water-insoluble steroid used during pars plana vitrectomy to make the transparent vitreous and ILM more visible, which aids in creating a posterior vitreous detachment and in peeling the ILM. However, there is valid concern in using TA as it can accumulate at the edge of the macular hole or straddle the hole edges, thereby inhibiting closure either as a result of mechanical interference or as an inhibitor of inflammation leading to a postoperative open hole and failure, though scattered case reports have also shown that residual TA in MHS does not seem to interfere with anatomic or visual improvement.[3]

## Case Report

**Figure 1:**
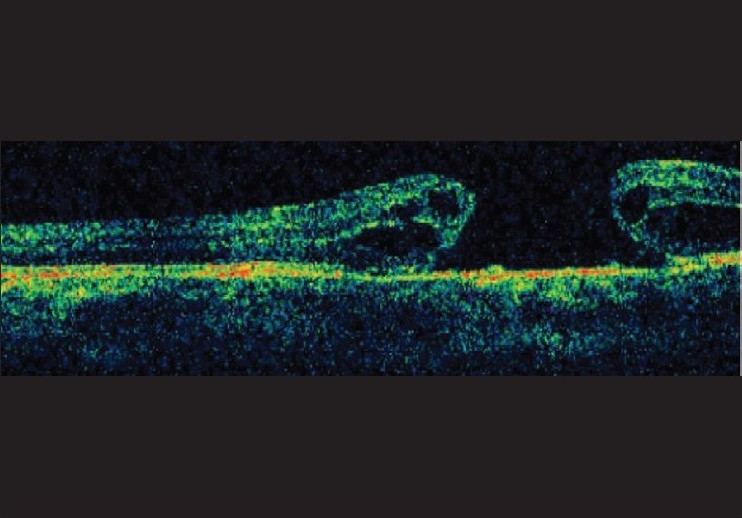
Right eye OCT line scan reveals open stage-IV hole

A 12-year-old boy presented with alleged history of sudden painless diminution of vision in his left eye following trauma with a soft ball of six weeks duration. The patient had a best corrected visual acuity (BCVA) of 20/200. On slit lamp biomicroscopy there was no sign to suggest anterior segment trauma. Optical Coherence Tomography (OCT) (Stratus OCT, version 4, Carl Zeiss) revealed a full thickness macular hole [Fig. 1] and Indirect Ophthalmoscopy showed associated infero-temporal multiple breaks with attached retina. The peripheral breaks were first treated with diode laser using a laser indirect ophthalmolscope (LIO) delivery. Two weeks later, macular hole surgery was carried out under general anesthesia after taking an informed consent. Posterior vitreous detachment induction was facilitated with TA crystals (preservative free), subsequent to which ILM peeling was performed using an ILM forceps (DORC, Zuidland, Netherlands). Fluid- air exchange was then carried out, followed by a 25% SF6 gas flush through the vitreous cavity. Post operative positioning was maintained for three days only.[4] One week post-surgery, the patient's BCVA improved to 20/125 with fundus and OCT images of the operated eye revealing residual TA in the foveal region at the macular hole site; these crystals exhibiting a hyper reflective mass corresponding to TA crystals on OCT [Fig. 2a and 2b]. On subsequent follow-up at one month post-surgery, OCT image showed disappearance of TA crystals with recovery of foveal morphologic features to an almost normal depression with hole closure [Fig. 3a and 3b]. Vision recovered to 20/80 and no intra ocular pressure elevation was observed throughout the observation period

**Figure 2a:**
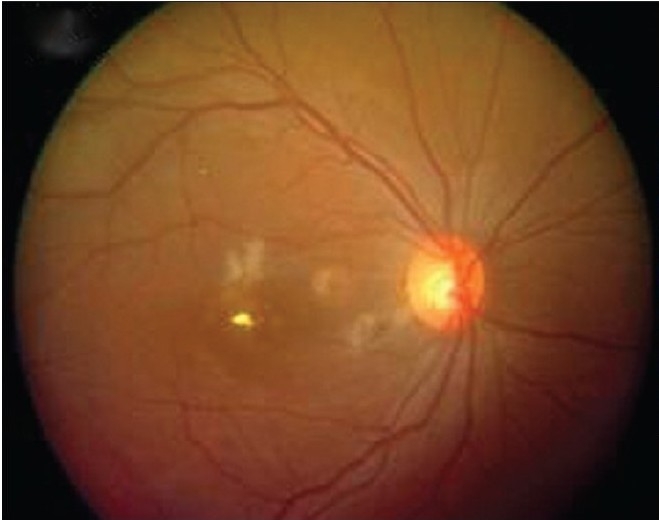
Colour fundus picture shows Triamcinolone Crystals caught within the hole at one week postop

**Figure 2b:**
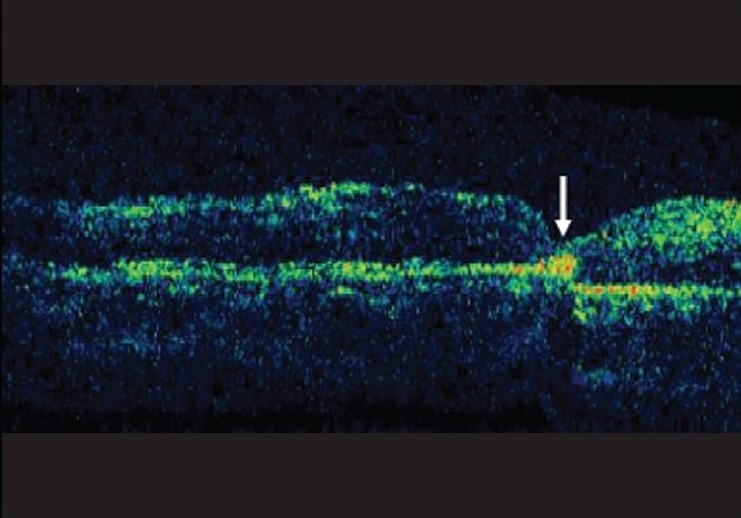
Arrow highlights hyper reflective crystals on OCT

**Figure 3a:**
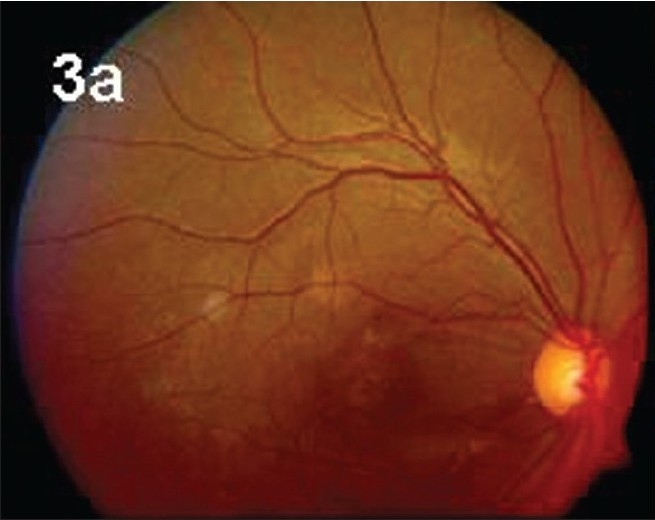
Closed hole on fundus picture at 4 week post-operatively

**Figure 3b:**
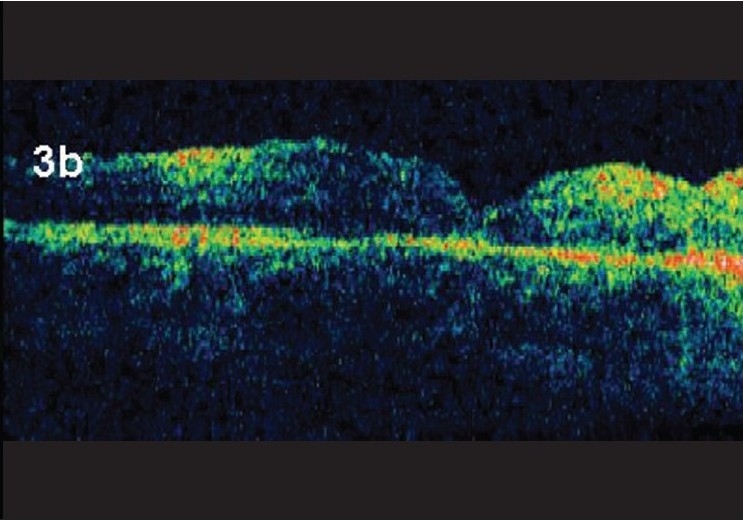
Line scan reveals closed hole with absence of crystals at four weeks post op

## Discussion

Intraocular use of TA is gaining popularity in patients for visualization of the vitreous cortex, PVD induction and ILM peeling during macular hole surgery. However, the possibility of residual steroid crystals clogging the hole at the conclusion of surgery exists.

A previous report suggested that a macular hole may not close completely and could reopen if TA crystals remain within it.[5] A number of theories may explain the underlying mechanism leading to the reopening of the macular hole. This may be attributable to a mechanical block by the TA crystals of the physiological interactions between the sensory retina and the retinal pigment epithelium (RPE). Alternatively, it may have been caused by the effect of TA as a corticosteroid because corticosteroids are known to alter the function of RPE cells.[6] A third possible reason might be, because the patient was young, the vitrectomy was performed under general anesthesia and therefore the patient was not moved to a facedown position for several hours, and the TA crystals within the vitreous cavity may have settled in the MH while the subject was in the face-up position immediately after surgery. However, conflicting outcomes exist in literature. The deposition of residual TA crystal on fovea after MHS has not been associated with poor visual outcome or interference with the closure of hole.[7,8]

Mahmoud and associates reported that foveolar lucencies observed by OCT were a common finding after macular hole surgery and that the findings gradually decreased and eventually resolved over time without additional surgical intervention and with further improvement of visual acuity.[9] In our case report, a young 12-year-old boy who sustained a traumatic stage 4 macular hole of size of 700μ closed despite poor compliance with postoperative positioning. We did not attempt to remove the residual TA in the macular hole during vitreous surgery. Although the TA was aspirated with a 23G soft silicon tipped cannula over the macular hole, we did not try to insert the vitrector or a silicon tipped cannula needle into the macular hole to remove the steroid crystals, as the procedure carries the risk of damaging the RPE on the floor of the macular hole, which may prevent the visual acuity from improving after closure of the macular hole. OCT image of the eyes with residual TA, observed in the fovea on the seventh day after surgery clinically, showed a hyper reflective mass corresponding to the TA on OCT. However, a repeat OCT carried out four weeks after surgery showed recovery of the foveal morphologic features to an almost normal depression, with closure of the hole.

Although the number of reported cases is too small to conclude that TA is non toxic to photoreceptor cells or the RPE, residual TA in the macular hole post vitreous surgery may not interfere with ultimate macular hole closure or visual improvement.
